# A phase I/II study of preoperative letrozole, everolimus, and carotuximab in stage 2 and 3 hormone receptor-positive and Her2-negative breast cancer

**DOI:** 10.1007/s10549-023-06864-9

**Published:** 2023-02-03

**Authors:** Christos Vaklavas, Erica M. Stringer-Reasor, Ahmed M. Elkhanany, Kevin J. Ryan, Yufeng Li, Charles P. Theuer, Edward P. Acosta, Shi Wei, Eddy S. Yang, William E. Grizzle, Andres Forero-Torres

**Affiliations:** 1 Huntsman Cancer Institute of the University of Utah, 2000 Circle of Hope, RS2509, Salt Lake, UT 84112 USA; 2 University of Alabama at Birmingham, Birmingham, AL USA; 3 TRACON Pharmaceuticals, San Diego, CA USA

**Keywords:** Letrozole, Everolimus, Carotuximab, Angiogenesis inhibitors, Breast cancer, Neoadjuvant therapy

## Abstract

**Purpose:**

In nonmetastatic hormone receptor-positive and Her2-negative breast cancer, preoperative endocrine therapies can yield outcomes similar with chemotherapy. We evaluated the tolerability and preliminary antitumor activity of preoperative letrozole, everolimus, and carotuximab, a monoclonal antibody targeting endoglin, in nonmetastatic breast cancer.

**Methods:**

Eligible patients had newly diagnosed, stage 2 or 3, hormone receptor-positive and Her2/neu-negative breast cancer. Patients received escalating doses of everolimus; the dose of letrozole and carotuximab were fixed at 2.5 mg PO daily and 15 mg/kg intravenously every 2 weeks, respectively. The primary objective was to determine the safety and tolerability of the combination. Secondary objectives included pharmacokinetic and pharmacodynamic studies and assessments of antitumor activity.

**Results:**

Fifteen patients enrolled. The recommended phase 2 dose of everolimus in combination with letrozole and carotuximab was 10 mg PO daily. The most frequent adverse events were headache (67%), fatigue (47%), facial flushing and swelling (47%), gingival hemorrhage (40%), epistaxis (33%), nausea and vomiting (27%). Headache constituted a dose-limiting toxicity. At least two signs of mucocutaneous telangiectasia developed in 92% of patients. Carotuximab accumulated in the extravascular space and accelerated the biodistribution and clearance of everolimus. All patients had residual disease. Gene expression analyses were consistent with downregulation of genes involved in proliferation and DNA repair. Among 6 patients with luminal B breast cancer, 5 converted to luminal A after one cycle of therapy.

**Conclusion:**

Letrozole, everolimus, and carotuximab were tolerated in combination at their single-agent doses. Pharmacokinetic studies revealed an interaction between everolimus and carotuximab.

**Trial registration:**

This trial is registered with ClinicalTrials.gov (Identifier: NCT02520063), first posted on August 11, 2015, and is active, not recruiting.

**Supplementary Information:**

The online version contains supplementary material available at 10.1007/s10549-023-06864-9.

## Introduction

In non-metastatic breast cancer, the achievement of a pathologic complete remission (pCR) with preoperative therapy has been consistently associated with better long-term outcomes[1, 2]. In hormone receptor-positive and Her2-negative (HR+/Her2−) breast cancer, preoperative chemotherapy and endocrine therapy, have been shown to yield similar benefits, albeit in unselected patients, with endocrine therapy being associated with significantly fewer adverse events[3]. Despite the high rates of clinical and radiographic responses with preoperative endocrine therapy[4–8], the rates of downstaging and more so pCR are still low [7].

Exploiting therapeutically the interplay between angiogenesis and estrogen receptor (ER), we have shown in 2 separate clinical trials that the addition of bevacizumab, a monoclonal antibody targeting the vascular endothelial growth factor (VEGF), to letrozole, leads to pCR rates (11–12%) comparable to preoperative chemotherapy [3, 9, 10]. The addition of mammalian Target of Rapamycin (mTOR) inhibitor to hormonal therapy reverses endocrine resistance and enhances the efficacy of hormonal agents [11–14]. mTOR inhibition abrogates VEGF-mediated signaling which, in turn, leads to compensatory overexpression of endoglin (CD105) in the endothelial cells [15, 16]. Upon VEGF inhibition, CD105(+) vessels in the periphery and core of tumors persist or regenerate [16]. Thus, the addition of an anti-CD105 agent may increase the antitumor efficacy seen in a randomized trial of preoperative letrozole and everolimus [12].

Endoglin (CD105) is a transmembrane accessory receptor for transforming growth factor-beta (TGF-β) that is predominantly expressed on proliferating endothelial cells and angiogenic blood vessels [17] as opposed to the normal quiescent endothelium which has a very low turnover [18, 19]. It diverts TGF-β downstream signaling toward proliferation and migration and promotes transcription of pro-angiogenic genes including endoglin itself [20–22]. Carotuximab (TRC105) is a chimeric immunoglobulin G1 monoclonal antibody that binds to endoglin, inhibits TGF-β mediated pro-angiogenic signaling, and induces potent antibody-dependent cell-mediated cytotoxicity[23]. In preclinical murine xenograft models of HR(+) breast cancer, anti-endoglin monoclonal antibodies selectively inhibited tumor angiogenesis and delayed the growth of established tumors, while sparing normal vasculature [24].

The purpose of the study was to determine the safety of combining everolimus and carotuximab with letrozole (with goserelin if premenopausal) in women with newly diagnosed stage 2 and 3 HR+/Her2− breast cancer. Secondary objectives included determination of the efficacy, pharmacokinetic and pharmacodynamic parameters of the triple combination. The study was terminated early because the clinical development of carotuximab was halted.

## Methods

### Patient eligibility

Eligible patients had newly diagnosed, potentially resectable, pathologically confirmed invasive stage 2 and 3 breast cancer (clinical T2, T3, T4a-c, N0-2, and M0), ER- and/or progesterone receptor (PR)-positive, Her2/neu-negative [25, 26]. At least 10% of tumor cell nuclei had to be immunoreactive for ER and/or PR. Breast magnetic resonance imaging (MRI) were performed at baseline, week 12 and 24. Patients with multicentric, multifocal, or bilateral breast cancer were allowed, so long as all separate tumors met eligibility criteria.

The protocol was amended once to allow premenopausal women whose ovarian function was to be suppressed with goserelin to participate in the study. The protocol was reviewed and approved by the participating institution, and the study followed the Declaration of Helsinki and Good Clinical Practice guidelines. All patients gave informed consent. This study is registered on the clinical trial website of the US National Cancer Institute (https://clinicaltrials.gov/ct2/show/NCT02520063).

### Study design and treatments

**Fig. 1:**
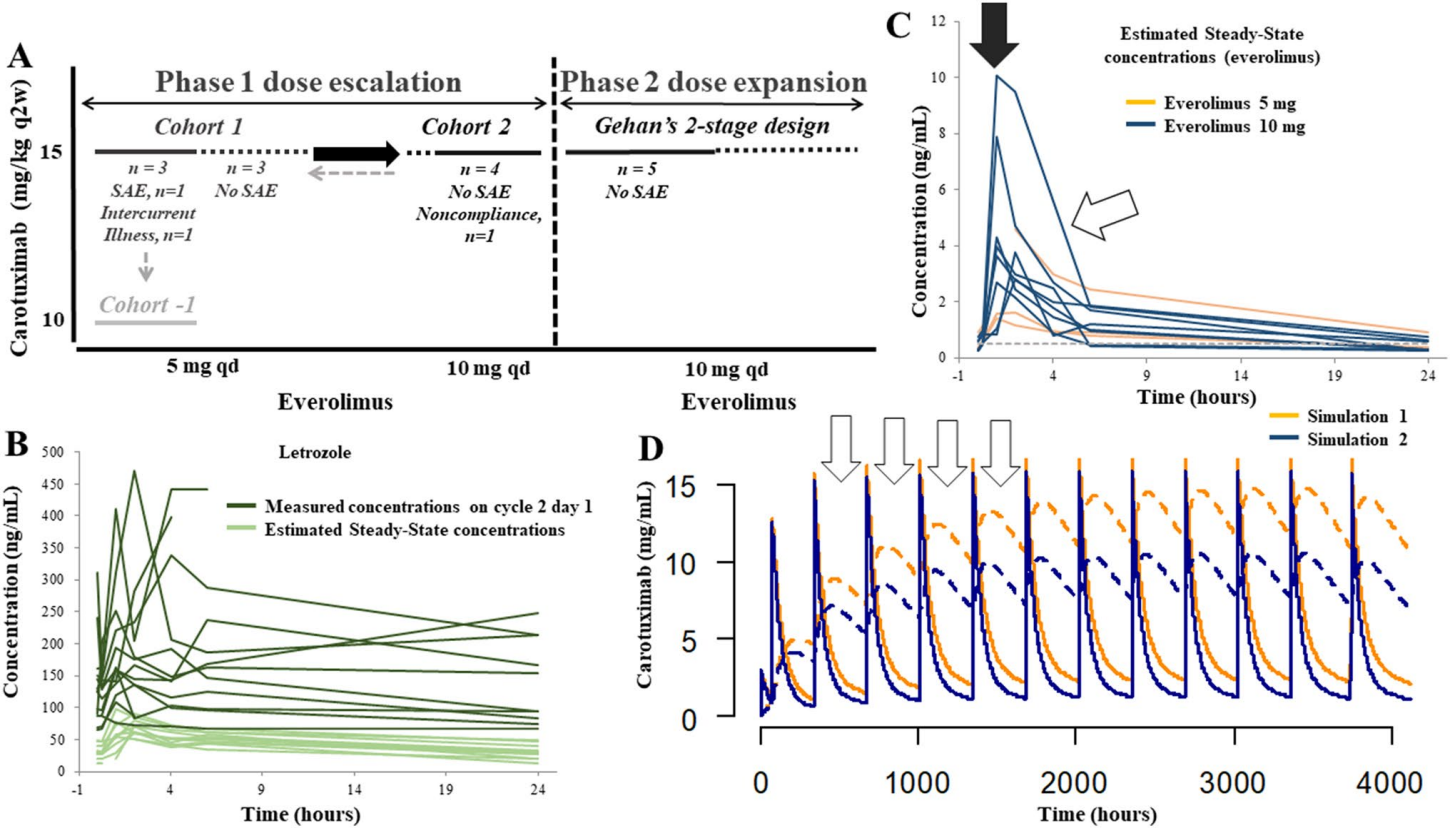
Study Schema and Pharmacokinetics. **A** The study had 2 parts (phase 1 and 2). Escalating doses of everolimus and carotuximab are shown in the *x* and *y* axis, respectively. The dose of letrozole and goserelin in premenopausal women was fixed in all cohorts. The thick black arrow indicates direction of dose escalation while the thin grey arrow indicates direction of de-escalation. The recommended phase 2 dose for everolimus and carotuximab was 10 mg PO daily and 15 mg/kg IV q 2 weeks, respectively. Five patients enrolled in the phase 2 part before the trial closed. (SAE, serious adverse event). **B** Spaghetti plot of the measured on cycle 2 day 1 and estimated steady-state concentrations of letrozole. Note the measured concentrations of letrozole being consistently higher than estimated indicative of accumulation and non-linear pharmacokinetic properties. **C** Spaghetti plot of the estimated steady-state concentrations of everolimus (color coded for dose). Note the lower than previously reported *C*max (solid arrow) and the rapid postabsorption distribution (empty arrow) of the 10 mg everolimus. **D** Simulated carotuximab concentration–time curves over the course of protocol therapy. Intravascular (continuous lines) and extravascular (dotted lines) concentrations color coded for 2 simulations: intravascular volume of 39 mL/kg, extravascular volume of distribution (137–39=) 98 mL/kg, intercompartmental clearance 27 mL/h/kg [59], clearance 0.321 (simulation 1) and 0.464 (simulation 2) [23] mL/kg/h. Note the rising concentrations of carotuximab in the extravascular space with successive administrations of carotuximab

This was a single-institution study with a dose escalation (phase 1) and expansion (phase 2) part (Fig. 1A). During the first part, the Maximum Tolerated Dose (MTD) and the Recommended Phase 2 Dose (RP2D) of the combination was determined. The dose of everolimus was escalated from 5 (cohort 1) to 10 mg PO daily (cohort 2). Everolimus was to be de-escalated to 5 mg PO daily (cohort 1) if 10 mg PO daily was not well tolerated. The dose of carotuximab was 15 mg/kg IV q 2 weeks (cohorts 1 and 2). Dose calculations were capped to a weight of 85 kg. The dose of carotuximab was to be de-escalated to 10 mg/kg IV q 2 weeks (cohort −1) if 15 mg/kg IV q 2 weeks in combination with everolimus 5 mg PO daily were not well tolerated. The dose of letrozole was fixed (2.5 mg PO daily). Premenopausal women received goserelin 3.6 mg subcutaneously q 4 weeks. Ovarian function suppression was monitored with estradiol levels on cycle 2 day 1 and q 2 cycles thereafter.

Letrozole was continued until the day prior to surgery. For everolimus and carotuximab, an interval of 4 weeks between last dose and surgery was required. The first dose of carotuximab was split into two infusions, each administered over 4 h, on days 1 (3 mg/kg) and 4 (12 mg/kg or 7 mg/kg if dose de-escalation occurred). If well tolerated, all subsequent infusions were administered as a whole every 2 weeks; infusion time was step-wise reduced to a minimum of 90 min. Premedications for each infusion included acetaminophen 650 mg PO, methylprednisolone 100 mg IV, famotidine 20 mg and cetirizine 10 mg PO or IV.

Delays for up to 3, 7, 7, and 14 days for goserelin, letrozole, everolimus, and carotuximab, respectively, were allowed. If carotuximab were delayed for > 3 days, the first dose of carotuximab upon resumption had to be split into two doses: 3 mg/kg on day 1 and the remainder on day 4. Dose reductions down to 2.5 mg PO daily and 10 mg/kg q 2 weeks were allowed for everolimus and carotuximab, respectively.

### Assessments for safety and efficacy

Toxicity was assessed using the National Cancer Institute Common Terminology Criteria for Adverse Events version 4.03 (https://evs.nci.nih.gov/ftp1/CTCAE/CTCAE_4.03/CTCAE_4.03_2010-06-14_QuickReference_5x7.pdf). Patients underwent evaluation of response by physical examination and breast ultrasound (at the beginning of every cycle), and breast MRI. Patients with objective response or stable disease according to the Response Evaluation Criteria in Solid Tumors version 1.1 (RECIST [27]) continued the investigational therapy for a maximum of 24 weeks.

### Definition of dose-limiting toxicity, pharmacokinetic analyses, nanostring analyses, immunohistochemistry, statistical considerations

See Supplementary Material.

## Results

### Patient characteristics and disposition

Fifteen patients enrolled and received at least one cycle of the investigational therapy (Table 1). Two patients had bilateral and one multifocal breast cancer. The age range of the patients was broad with near-equal representation of pre- and postmenopausal women. All patients had ECOG performance status 0. The average primary tumor size on MRI was 5.01 cm.

**Table 1:** Patient and tumor characteristics

Patient characteristics (*n* = 15)	
Age	
Median (range)	56 (29–70)
Race [*n* (%)]	
White	12 (80)
Black	3 (20)
Menopausal status [*n* (%)]	
Premenopausal	7 (47)
Postmenopausal	8 (53)
Tumor characteristics (*n* = 17)^a^	
Clinical stage [*n* (%)]	
IA	1^b^ (6)
IIA	7 (41)
IIB	2 (12)
IIIA	7 (41)
Nodal status at diagnosis [*n* (%)]	
N0	9 (53)
N1	5 (29)
N2a	2 (12)
N2b	1 (6)
Histologic type [*n* (%)]	
Invasive ductal carcinoma	10 (59)
Invasive lobular carcinoma	4 (24)
Invasive mammary carcinoma	3 (17)
Histologic Grade (modified Bloom Richardson scale) [*n* (%)]	
Grade 1	2 (12)
Grade 2	11 (64)
Grade 3	2 (12)
Not reported	2 (12)

^a^Two patients had bilateral and one multifocal breast cancer

^b^This patient had contralateral stage IIIA breast cancer

### Maximum tolerated dose and dose-limiting toxicities

Ten patients enrolled in the phase 1 part (Fig. 1A). Among the first 3 patients enrolled in cohort 1 (everolimus 5 mg PO daily + carotuximab 15 mg/kg q 2 weeks), one patient discontinued permanently protocol therapy due to intercurrent illness unrelated to protocol therapy (MediPort infection) and one patient had a serious adverse event (“first-dose” headache [see below]). She resumed treatment with 1 week delay and completed protocol therapy without dose reductions or further interruptions. Cohort 1 was expanded to a total of 6 patients; no serious adverse events were noted and the trial proceeded to cohort 2 (everolimus 10 mg PO daily + carotuximab 15 mg/kg q 2 weeks). Four patients enrolled in cohort 2 to account for one patient who was noncompliant and taken off study. No serious adverse events were seen in cohort 2. The RP2D was declared at letrozole 2.5 mg and everolimus 10 mg PO daily + carotuximab 15 mg/kg q 2 weeks ± goserelin 3.6 mg SC q 4 weeks; the MTD was not reached.

### Safety and tolerability

All patients experienced adverse events (Table 2). The nature and frequency of adverse events were similar in the two phase I cohorts. The majority of adverse events were mild to moderate in severity (grade 1–2). The profile of adverse events of the combination was dominated by the ones associated with carotuximab [23, 28–32]. Adverse events typically associated with aromatase inhibitors (hot flashes, joint stiffness) and metabolic adverse events typically associated with everolimus (dyslipidemias) arose after cycle 1 and were comparatively less frequent.

**Table 2:** Adverse events classified by time of occurrence (cycle 1 vs. cycle 2–6) in decreasing order of frequency

	Number of patients (percent)			
	Cycle 1 (MTD period) (*n* = 15)		Cycles 2 through 6 (*n* = 12)^a^	
	Any grade	Grade ≥ 3	Any grade	Grade ≥ 3
Adverse event				
Headache	10 (67)	2 (13)	11 (92)	0
Fatigue	7 (47)	0	12 (100)	0
Facial flushing and swelling	7 (47)	0	6 (50)	0
Gingival hemorrhage	6 (40)	0	11 (92)	0
Epistaxis	5 (33)	0	12 (100)	0
Nausea and vomiting	4 (27)	0	1 (8)	0
Nasal congestion	3 (20)	0	8 (67)	0
Rash	3 (20)	0	9 (75)	0
Mucositis, oral ulcers, oral soreness	3 (20)	0	5 (42)	0
Telangiectasias	2 (13)	0	3 (25)	0
Gingival tenderness	1 (7)	0	3 (25)	0
Dysgeusia	1 (7)	0	1 (8)	0
Insomnia	1 (7)	0	1 (8)	0
Tingling, numbness	1 (7)	0	2 (17)	0
Diarrhea	1 (7)	0	2 (17)	0
Depression	1 (7)	0	0	0
Shaking chills and sweats	1 (7)	0	1 (8)	0
Constipation	0	0	1 (8)	0
Weight loss	0	0	1 (8)	0
Nail discoloration	0	0	1 (8)	0
Dyspnea	0	0	2 (17)	0
Hot flashes	0	0	1 (8)	0
Joint stiffness	0	0	4 (33)	0
Edema	0	0	1 (8)	0
Lab abnormalities				
Hyperglycemia	1 (7)	0	1 (8)	0
Anemia	0	0	4 (33)	0
Hypercholesterolemia	0	0	2 (17)	0
Hypertriglyceridemia	0	0	2 (17)	0

^a^Three patients were taken off study after completion of cycle 1 due to intercurrent illness, noncompliance, progressive disease (each, *n* = 1)

At least two signs of mucocutaneous telangiectasia (epistaxis, gingival bleeding, and telangiectasia), that typify hereditary hemorrhagic telangiectasia (HHT) were seen in 40% (6/15) of patients during cycle 1 and 92% (11/12) of patients who continued on the investigational therapy beyond cycle 1. HHT1 or Osler–Rendu–Weber syndrome 1 is a genetic disorder characterized by a deficiency in endoglin. Headache was the most frequent adverse event during cycle 1 and constituted the only dose-limiting toxicity (DLT). As previously reported [32], headache was not associated with hypertension or focal neurologic signs. In the patient in whom imaging was performed, no radiographic abnormalities were seen. “First-dose” headache has been mitigated by splitting the first dose of carotuximab in 2 separate infusions [32], a strategy that was adopted in our trial. Although everolimus has also been associated with headache (11% when used in combination with letrozole [12]), headache was temporally associated with the infusions of carotuximab.

Although the adverse events were mostly grade 1 or 2, they all tended to persist, accumulate, and worsen with subsequent cycles of therapy. These adverse events were most notably fatigue which all patients beyond cycle 1 experienced; normocytic normochromic hypoproliferative anemia suspected to result from carotuximab’s effect on the proerythroblasts [23, 33]; epistaxis which would be triggered by progressively milder stimuli or even occur spontaneously. Although gingival hemorrhage is a distinctive, “on-target” adverse event associated with carotuximab, the higher frequency with which gingival hemorrhage was seen in this trial (92% vs. 31–50% [28, 29, 32]) alongside with gingival tenderness and mucositis may reflect a synergistic effect between everolimus and carotuximab. Similarly, a synergistic effect between carotuximab and everolimus may underpin the universal occurrence of fatigue (reported with both everolimus and carotuximab), and the high frequency of skin rash (typically associated with everolimus, reported in 20% of patients [12] vs. 75% in this study).

Toxicities associated with VEGF inhibition (hypertension, proteinuria, thrombosis) and serious infusion reactions were not seen. The dose of everolimus was reduced in 4 patients due to rash (*n* = 2, dose reduction within the first cycle) and fatigue (*n* = 1, dose reduction in cycle 4); in one patient the reason for dose reduction on cycle 6 day 15 was not reported. Carotuximab was dose reduced in 1 patient on cycle 4 day 15 due to gingival hemorrhage. Eleven patients (73%) completed protocol therapy. Reasons for discontinuation included (all, *n* = 1) intercurrent illness, noncompliance, progressive disease (all taken off study on cycle 2); and development of anasarca/generalized edema (taken off study on cycle 4; association with the investigational therapy could not be determined).

### Pharmacokinetics

Letrozole was rapidly absorbed from the gastrointestinal tract (maximum concentration (Cmax) reached within 2 h). With repeated daily administration, Cmax and the area under the concentration–time curve (AUC) increased by 5.5- and 2.4-fold, respectively, consistent with accumulation [34] (supplementary table 1). The measured concentrations of letrozole (which reaches steady state in 2–6 weeks [35]) on cycle 2 day 1 were consistently higher and more variable as compared with the predicted concentrations indicating non-linear pharmacokinetics (Fig. 1B).

PK analyses of everolimus were consistent with significantly lower maximum and trough concentrations (Ctrough) from those previously reported (Cmax: 5 mg, 2.63 ng/mL; 10 mg, 5.38 ng/mL vs. 32 and 61 ng/mL, respectively [36]; Ctrough: 5 mg, 0.354 (estimated) ng/mL; 10 mg, 0.323 (estimated) ng/mL vs. 5.4 and 13.2 ng/mL, respectively [36], Fig. 1C and supplementary table 1). Similarly, the AUC’s at steady state were calculated to be at 1/10 of the AUC’s previously reported [36, 37]. The half-life of everolimus was similar in the 2 dose levels (5 mg: 13.4 h, 10 mg: 11.8 h), and significantly lower from the 30 h previously reported [36]. There was a good concordance between the estimated and actual steady-state concentrations (supplementary Fig. 1A). No PK interactions between letrozole and everolimus are known to occur [37] indicating that carotuximab accelerated significantly the extravasation of everolimus (estimated volume of distribution exceeded 4000 and 5000 L for 5 and 10 mg of everolimus, respectively) as well as its clearance to the point that predose everolimus concentrations dropped below the lower limit of quantitation.

Serum concentrations of carotuximab known to saturate CD105 receptors (200 ng/mL) were rapidly achieved and consistently maintained (supplementary Fig. 1B). With dose escalation from 3 to 15 mg/kg, AUC and half-life increased supraproportionally while clearance decreased by a similar magnitude probably due to target saturation (supplementary table 2). PK simulations using a two-compartment model suggest accumulation of carotuximab in the extravascular volume of distribution (Fig. 1D).

### Pharmacodynamics and antitumor activity

**Fig. 2:**
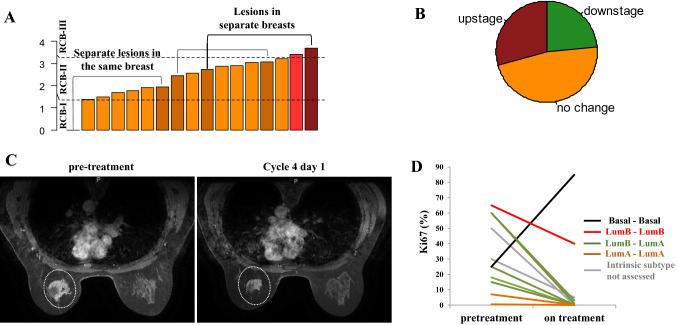
Antitumor activity. **A** Barplot of the RCB scores for the individual tumors. The patient with basal-like intrinsic subtype who transitioned to chemotherapy was excluded. **B** Piechart of change in stage (clinical stage at diagnosis – pathologic stage at surgery) with the investigational regimen. **C** Representative imaging response on MRI. **D** Pre- and on-treatment (cycle 2 day 1) changes of Ki67. Note the upregulation of Ki67 in the patient with the basal-like intrinsic subtype

All patients underwent definitive surgery with curative intent. One patient with a basal-like intrinsic molecular subtype (see below) progressed clinically and radiographically and she transitioned to preoperative chemotherapy. All patients had residual disease on surgical pathology; pCR was achieved in one of two sites in a patient with multifocal disease. To capture treatment effect, we also assessed pathologic response by means of Residual Cancer Burden (RCB) scoring [38] (Fig. 2A). Among the remaining tumors, moderate treatment effect (RCB-II) was seen in 14 (14/16, 87.5%; 95% CI 61.65–98.45) while residual disease was extensive in 2 (RCB-III, 12.5%; 95% CI 1.55–38.35). Downstaging from stage 2/3 to 0/1 was achieved in 4/17 tumors (23.5%; 95% CI 6.81–49.9), stage did not change in 8/17 (47.1%; 95% CI 23–72.2), and 4/17 tumors were upstaged (23.5%; 95% CI 6.81–49.9) (Fig. 2B). Of the 4 tumors that were upstaged, 3 were invasive lobular and 1 invasive mammary carcinomas. Given the diffuse growth pattern and lack of desmoplastic reaction in invasive lobular carcinoma, this finding may reflect underestimation of the extent of the original disease rather than interval tumor growth [10]. Representative radiographic responses are shown in Fig. 2C and supplementary Fig. 2. The expression of the proliferation marker Ki-67 also decreased with the investigational therapy and its changes aligned with intrinsic subtype changes or lack thereof (Fig. 2D).

To characterize gene expression changes induced by the investigational therapy, we performed genomic analyses on a NanoString nCounter platform (Breast Cancer 360 panel) of paired macrodissected tumor samples obtained at the time of diagnosis and on cycle 2 day 1. We successfully tested and analyzed 10 paired samples; one on-treatment sample did not pass quality control.

**Fig. 3:**
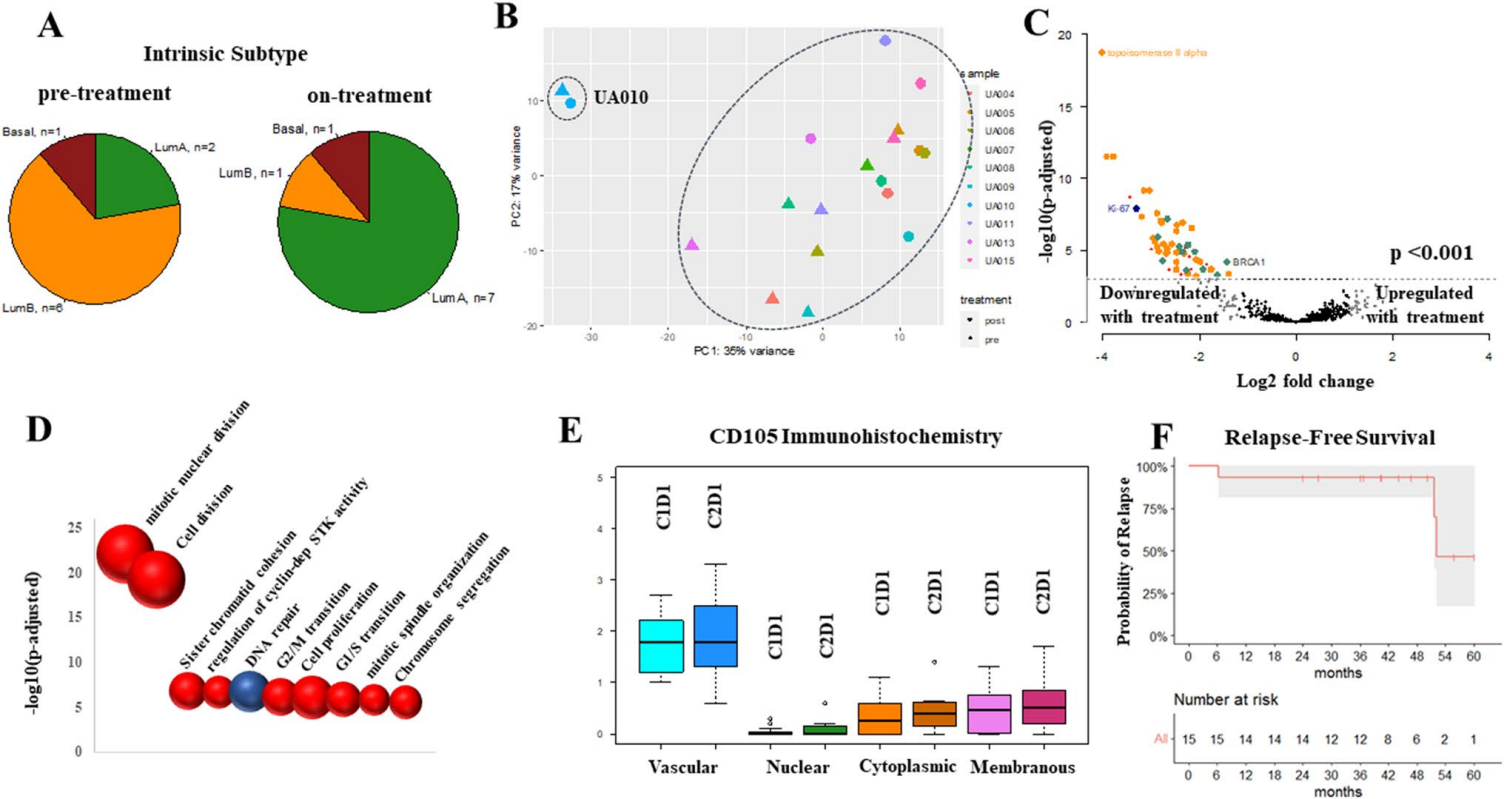
Correlative Studies and Relapse-free Survival. **A** Distribution of the intrinsic molecular subtypes at diagnosis and after one cycle of the investigational therapy. **B** Principle component analysis of all samples (pre- and on-treatment) with successful gene expression profiling by Nanostring. Note the distinction of the tumor with the basal-like intrinsic subtype from the cluster with the luminal tumors. Note also the proximity of the pre (circle) and on-treatment (triangle) points for the basal-like intrinsic subtype suggestive of lack of changes in gene expression with treatment unlike the luminal counterparts. **C** Volcano plot of the differentially expressed genes in the luminal cancers following one cycle of the investigational therapy. Differentially expressed genes at *p*-adjusted < 0.001 (above dashed horizontal line) are indicated in red; differentially expressed genes at *p*-adjusted < 0.001 involved in cell proliferation and DNA repair indicated in darkorange and aquamarine, respectively. **D** Bubble plot of the ontology of genes whose expression was downregulated with treatment *p* < 0.001 (adjusted by the Bergamini–Hochberg method for multiple comparisons). Bubbles are color coded by ontology category. The size of each bubble is proportionate to the number of genes falling into this category. Gene ontologies are plotted against the significance of gene-term enrichment (*p*-value, *y*-axis). **E** Immunohistochemistry for CD105 assessed in the vascular, nuclear, cytoplasmic, and membranous compartment on cycle 1 day 1 (C1D1) and cycle 2 day 1 (C2D1). **F** Kaplan–Meier curve of the relapse-free survival

By *PAM50* Molecular Subtype Signature, at diagnosis, 6 patients had luminal B, 3 patients had luminal A, and 1 patient had basal-like intrinsic subtype (Fig. 3A, supplementary Fig. 3 and 4). Of the 6 patients with luminal B breast cancer at diagnosis, 5 converted to luminal A on cycle 2 day 1 yielding a “molecular downstaging” rate of 83.3% [39]. Excluding the patient with the basal-like intrinsic subtype who had a distinctive gene expression profile (Fig. 3B and supplementary Fig. 5A), differential gene expression analyses between paired pre- and on-treatment samples show significant downregulation in the expression of genes involved in or regulating cell division (39/53 genes significantly downregulated at a p adjusted for multiple comparisons by Bergamini–Hochberg < 0.001; enrichment score p 8.89 × 10^–23^/False Discovery Rate 2.73 × 10^–22^) (Fig. 3C, D). The second most overrepresented functional category of downregulated genes, included genes involved in DNA repair most notably BRCA1 (enrichment score p 2.08 × 10^–7^/False Discovery Rate 3.21 × 10^–6^). A possible explanation for the upregulation and attendant treatment-related downregulation of these genes, may rely on fact that the ER induces transcriptional stress via R-loop formation, i.e. 3-stranded nucleic acid structures comprising a DNA:RNA duplex and a displaced single-stranded DNA [40]. Persistent R-loop accumulation can compromise genomic integrity which in turn, is mitigated by the *BRCA1/2* genes [41, 42].

The presence of non-luminal intrinsic subtypes in the immunophenotypically HR+/Her2− subgroup has been well recognized [43, 44]. By comparison to the luminal tumors, overexpression of genes that typify a basal-like subtype (*cytokeratin 5* [45, 46], *BCL11A* and *FOXC1* [47, 48]) or are associated with resistance to endocrine therapies (*STAT1* [49], *cyclin E* [50]), and the lower expression of *ESR1* and *PGR* may underpin the absence of gene expression changes and clinical response to the investigational therapy (supplementary Fig. 5 A and B).

CD105 (endoglin) was predominantly expressed in the vessels (Fig. 3E). CD105 expression, as assessed by immunohistochemistry, did not change significantly with the investigational therapy. The lack of change in CD105 expression may be due to early on-treatment CD105 evaluation because decreases in signal enhancement between baseline and cycle 4 day 1 breast MRIs were observed (Fig. 2C and supplementary Fig. 2). The absence of change may also be consistent with the mechanism of action of carotuximab whereby the antibody accelerates the cleavage and shedding of endoglin by coupling it with the membrane-anchored matrix-metalloproteinase 14 without changing the expression of the endoglin itself [51].

Semiquantitative assessment of the residual tumors for stromal tumor infiltrating lymphocytes (TILs) was consistent with no or minimal levels of TILs in 15/17 tumors (patient who progressed and transitioned to chemotherapy excluded; 88.2%; 95% CI 63.6–98.5) [52, 53].

The protocol left postoperative treatment decisions at the discretion of the treating physicians. None of 4 patients whose tumors downstaged with the investigational therapy received adjuvant chemotherapy; 4/7 (57%) and 2/3 (66%) patients whose tumors did not change stage or were upstaged, respectively, received adjuvant chemotherapy. With a median follow up of 45.6 months after surgery for curative intent (53.4 months from cycle 1 day 1), 3 patients relapsed (Fig. 3F, supplementary Fig. 6). The patient with the basal-like intrinsic subtype experienced an early recurrence despite neoadjuvant chemotherapy. Among the 5 patients whose tumors converted from luminal B to luminal A, 2 received adjuvant chemotherapy. None of the remaining 3 patients experienced a recurrence (follow up, 24.1–50.4 months; median, 40.1).

## Discussion

This study met its primary endpoint of determining the MTD, RP2D, and rates of adverse events associated with the combination of letrozole with everolimus and carotuximab ± goserelin. All agents were tolerated at their single-agent doses. The toxicity profile was dominated by carotuximab and phenocopied HHT. Despite the paucity of adverse events greater than grade 2, all patients experienced adverse events that accumulated over time leading to frequent dose reductions. One should acknowledge the excellent performance status of patients with newly diagnosed breast cancer as well as the fact that endocrine combinations with novel oral agents, particularly inhibitors of the cyclin dependent kinase 4 and 6 (CDK4/6), can yield similarly high rates of molecular downstaging with fewer toxicities [39].

Pharmacokinetic analyses, which constituted a secondary objective of the study, show that, despite the targeted nature of carotuximab and everolimus, interactions did arise. The accumulation of carotuximab in the extravascular space with successive administrations may explain the later-onset anemia (typically seen after cycle 3) and the rising frequency and severity of adverse events associated with carotuximab (headaches, fatigue, epistaxis, gingival hemorrhage, nasal congestion, and telangiectasias). Carotuximab also accelerated the biodistribution of everolimus which probably underpins a pharmacodynamic interaction, in terms of significant downregulation of a proliferative gene expression signature as well as causation of synergistic adverse events (fatigue, skin rash, gingival hemorrhage). The long interval required for letrozole to reach steady state and the accumulation with repeated dosing may explain the later-onset of adverse events typically associated with this agent.

In terms of assessing the antitumor activity of preoperative endocrine therapy and especially combinatorial regimens, uniform criteria for optimal patient selection, endpoints to capture the activity of preoperative endocrine therapy, and biomarkers to escalate or de-escalate therapy have not been adopted.

Our results and other trials [54] suggest that molecular criteria should be considered for patient selection in these trials. Luminal A tumors may respond to endocrine monotherapy; additional agents and chemotherapy can be spared [55]. Nonluminal HR+/Her2− tumors and especially basal-like have distinct molecular underpinnings and a response to endocrine therapy should not be expected [44]. In our study, the most meaningful reductions in proliferation and gene expression changes were seen in luminal B tumors.

Recognizing that the achievement of pCR is an endpoint that does not align with the nature of luminal breast cancer and conventional pathologic criteria do not capture the gene expression changes induced by preoperative endocrine therapy, other, mostly proliferation-based biomarkers have been used [44, 53, 56–58]. Reductions in Ki67 with preoperative endocrine therapy have been associated with excellent long-term outcomes sparing chemotherapy [44, 54]. However, how well reductions in Ki67 can prognosticate patients with higher genomic risk disease who receive preoperative combinatorial endocrine regimens remains an open question: postoperative treatment decisions were left to the physicians’ discretion, long-term outcomes have not been reported or captured, and Ki67 can rebound upon treatment discontinuation [53, 57]. Regarding MammaPrint, the appealing concept of molecular downstaging, i.e. conversion from luminal B to PAM50 low-risk-of relapse disease, has been introduced [39]. Its ability to predict relapse or identify patients who can safely forego adjuvant chemotherapy and, more so, whether it has the same value irrespective of the regimen that induced it, remain to be evaluated. Although the administration of postoperative chemotherapy at physicians’ discretion precludes a clean readout of the long-term impact of the investigational therapy, our results provide clues that patients who achieve molecular downstaging have low risk of early relapse and potentially can be spared chemotherapy.

In conclusion, letrozole, everolimus, and carotuximab ± goserelin were tolerated in combination at their single-agent doses. Escalating the number of targeted agents in combinatorial preoperative regimens, may enhance the toxicities due to unaccounted interactions while the respective increments in antitumor activity may remain elusive.

## Supplementary Information

Below is the link to the electronic supplementary material.

Supplementary file1 (DOCX 38 kb)

Supplementary file2 (PPTX 102 kb)

Supplementary file3 (PPTX 38056 kb)

Supplementary file4 (PPTX 879 kb)

Supplementary file5 (PPTX 970 kb)

Supplementary file6 (PPTX 141 kb)

Supplementary file7 (PPTX 52 kb)

Supplementary file8 (DOCX 15 kb)

Supplementary file9 (DOCX 14 kb)

Supplementary file9 (DOCX 28 kb)

## Data Availability

The datasets generated during and/or analyzed during the current study are available in the Mendeley repository (https://data.mendeley.com/datasets/kxyb98kynn/draft?a=fc195a30-f79c-48df-8006-9fc9e69f7474).

## Abbreviations

AUC: Area under the concentration–time curve
BCL11A: BAF chromatin remodeling complex subunit BCL11A
BRCA1: BRCA1 DNA repair associated
BRCA2: BRCA2 DNA repair associated
CD105: Endoglin
CDK4/6: Cyclin-dependent kinase 4 and cyclin-dependent kinase 6
C1D1: Cycle 1 day 1
C2D1: Cycle 2 day 1
CI: Confidence interval
Cmax: Maximum concentration
Ctrough: Trough concentration
DLT: Dose-limiting toxicity
DNA: Deoxyribonucleic acid
ECOG: Eastern cooperative oncology group
ER/ESR1: Estrogen receptor
FOXC1: Forkhead box C1
Her2: Epidermal growth factor receptor-2
HHT: Hereditary hemorrhagic telangiectasia
HR: Hormone receptor
IV: Intravenous
kg: Kilogram
mg: Milligram
µL: Microliter
MRI: Magnetic resonance imaging
MRM: Multiple reaction monitoring
MTD: Maximum tolerated dose
mTOR: Mammalian target of rapamycin
ng: Nanogram
pCR: Pathologic complete remission
PK: Pharmacokinetic
PO: Per os (oral)
RCB: Residual cancer burden
PR/PGR: Progesterone receptor
RECIST: Response evaluation criteria in solid tumors
RNA: Ribonucleic acid
RP2D: Recommended phase 2 dose
SC: Subcutaneously
SAE: Serious adverse event
STAT1: Signal transducer and activator of transcription 1
TGF-β: Transforming growth factor-beta
TILs: Tumor infiltrating lymphocytes
TRC105: Carotuximab
VEGF: Vascular endothelial growth factor
vs.: Versus
