# Supplementary material for: A phase I/II study of preoperative letrozole, everolimus, and carotuximab in stage 2 and 3 hormone receptor-positive and Her2-negative breast cancer
Source: Breast Cancer Res Treat. 2023 Feb 3;198(2):217–29. doi: 10.1007/s10549-023-06864-9 (PMC10020303; doi:10.1007/s10549-023-06864-9)
Supplement: Supplementary file 1 — Supplementary file1 (DOCX 38 kb) [file 10549_2023_6864_MOESM1_ESM.docx]

**Supplementary Material**

**Title:** A Phase I/II Study of Preoperative Letrozole, Everolimus, and Carotuximab in Stage 2 and 3 Hormone Receptor-positive and Her2-negative Breast Cancer.

**Running Head**: Letrozole, everolimus, and TRC105 in Breast Cancer

**Authors:** Christos Vaklavas^1,2^ (ORCID, 0000-0002-9919-2748), Erica M. Stringer-Reasor^2^ (ORCID, 0000-0002-1862-3367), Ahmed M Elkhanany^2^ (ORCID, 0000-0001-7681-6872), Kevin J Ryan^2^, Yufeng Li^2^, Charles P Theuer^3^ (ORCID, 0000-0003-2826-4051), Edward P Acosta^2^, Shi Wei^2^, Eddy S Yang^2^ (ORCID, 0000-0002-6450-2638), William E. Grizzle^2^ (ORCID, 0000-0001-9245-0401), Andres Forero-Torres^2^ (ORCID, 0000-0001-5161-8319)

**Institutions**: ^1^Huntsman Cancer Institute of the University of Utah, UT, USA; ^2^University of Alabama at Birmingham, Birmingham, AL, USA; ^3^TRACON Pharmaceuticals, USA*.*

**Correspondence Information:** Christos Vaklavas, M.D.; 2000 Circle of Hope, RS2509 | Huntsman Cancer Institute of the University of Utah | Salt Lake City, UT 84112 | phone: 801 213 8477, fax: 801 587 8143 | [christos.vaklavas@hci.utah.edu](mailto:christos.vaklavas@hci.utah.edu)

**Supplementary Methods**

***Definition of Dose Limiting Toxicity***

Dose limiting toxicity (DLT) was defined as: (1) any grade 3 or 4 non-hematologic toxicity except anorexia, alopecia, nausea (not refractory to antiemetics), fatigue, fever without neutropenia; (2) failure to recover to baseline (except alopecia) after delaying the next dose by more than 14 days; (3) grade ≥3 neutropenia complicated by fever >38.5°C or infection, or grade 4 neutropenia of ≥7 days duration; (4) grade 4 thrombocytopenia, or grade 3 thrombocytopenia complicated by hemorrhage. The DLT period was defined as the first cycle.

***Pharmacokinetic Analyses***

Serum samples were collected on cycle 1 day 1 and cycle 2 day 1 at the following time points: pre-dose, 1, 2, 4, 6, and 24 hours; serum samples were also collected on cycle 1 day 4 pre- and post-dose and cycle 1 day 15 post-dose.

Letrozole and Everolimus were quantified with Liquid-Liquid Extraction and high-performance liquid chromatography with tandem mass spectrometry detection (LC-MS/MS). Labeled Letrozole (^2^H_4_) and Everolimus (^2^H_4_) were used as the internal standards (Letrozole-d_4_ and Everolimus-d_4_, respectively). Letrozole and Letrozole-d_4_ were extracted from 100 µL human serum using LLE with ethyl acetate. Everolimus and Everolimus-d_4_ were extracted from 100 µL human serum using protein precipitation with methanol. Extracts were analyzed by reverse-phase chromatography using an ACE Excel 5 C18-PFP column under gradient conditions at a flow rate of 500 µL/minute. The column temperature was maintained at 40°C. The mobile phases consisted of 0.1% formic acid in water for mobile phase A and is 0.1% formic acid in methanol for mobile phase B. A triple quadrupole mass spectrometer (AB Sciex 5000) equipped with TurboV IonSpray^®^ operating in negative-ion (letrozole) or positive-ion (everolimus) mode was used. Column effluents were analyzed by multiple reaction monitoring (MRM). The precursor/product transitions were 284.1→242.3 m/z for Letrozole, 288.1→246.5 m/z for Letrozole-d_4_, 975.7→908.1 m/z for Everolimus, and 979.7→908.1 m/z for Everolimus-d_4_. The calibration curve was fit using weighted (1/x^2^) linear regression analysis of the Letrozole/Letrozole-d_4_ and Everolimus/Everolimus-d_4_ peak area ratio versus the Letrozole and Everolimus concentration, respectively, from 0.1 – 100 ng/mL. Concentrations of incurred and quality control samples were calculated with the same regression analysis and results were reported in ng/mL. The lower limit of quantitation were 0.1 ng/mL and 0.5 ng/mL for letrozole and everolimus, respectively.

Serum concentrations of carotuximab were determined during the phase 1 part using a validated ELISA with a limit of quantitation of 78 ng/mL as previously described [1, 2].The carotuximab used in the trial was produced in a Chinese hamster ovary (CHO) cell line that used media free of animal derived components. Neither Human anti-murine antibody (HAMA) nor Human anti-chimeric antibody (HACA) have been previously detected in patients treated with CHO-produced carotuximab[1].

Pharmacokinetic analyses were performed using R version 3.6.1 and the following packages “PK”, “PKNCA”, “PKPDsim”. Concentrations of letrozole and everolimus other than the cycle 1-predose timepoint that were below the lower limit of quantitation were assumed to be ½ * lower limit of quantitation.

***Nanostring Analyses***

Breast cores were frozen in OCT blocks, macrodissected by an expert pathologist (WEG), thawed and immediately put into TRIzol reagent (Ambion, Carlsbad, CA) for RNA isolation. After breaking up the tissue with a disposable pestle, they were passed through an 18 gauge needle to complete the homogenization. The TRIzol Plus RNA Purification Kit (Invitrogen, cat#1218355) protocol was used, as per manufacturer’s instructions. Briefly, choroform was added, the samples thoroughly mixed, and centrifuged for 15 min at 12000xg at 4C. The colorless aqueous phase was added to an equal volume of 70% ethanol, mixed well and added to a spin cartridge. After various washes, the samples were collected in 30 µL aliquots of RNase free water, and checked on a DeNovix DS-11 Spectrophotometer (Wilmington, DE) for A260/A280 and A260/A230 ratios. If the RNA concentration was less than 20mg/ µL, they were concentrated using RNA Clean & Concentrator-5 kit (Zymo Research) and eluted in 10 µL of RNase free water. RNA (100 ng) for each sample was hybridized with the Reporter CodeSet and Capture ProbeSet of the premade Breast Cancer IO360 panel (NanoString Technologies, Seattle, WA) overnight at 65C, and run on the NanoString Dx Prep Station. They were counted on the Dx Digitial Analyzer using an FOV of 555 (max) as specified by the manufacturer. Standards provided with the panel were also run as instructed for the BC360. Data Analysis Service performed by NanoString Technologies (molecular subtype classification); all other analyses were performed using R version 3.6.1.

***Immunohistochemistry***

The assay for Ki-67 and CD105 were performed using the rabbit monoclonal antibody MIB1 and recombinant rabbit monoclonal clone 103 (MA5-29234, Invitrogen), respectively. The Ki-67 assessment was performed by the clinical histology laboratory using a validated assay. The results were evaluated by a board certified pathologist (WEG), who estimated the proportion of cancer cells stained for Ki-67. The individual tissue sections were analyzed blindly and at the completion of the assay, results were correlated per case as to changes in Ki-67 and CD105 with therapy.

***Statistical Considerations***

The phase 1 part of the study followed a conventional 3+3 dose escalation design. The phase 2, expansion phase ensued once the MTD and RP2D were determined. A Gehan’s two-stage design was used[3]. If no pCR or downstaging among the first 10 patients were seen, the study would close on the basis that it was unlikely (p, 0.1074) that 0/10 responses would have occurred if the true response rate were >20%. If ≥1 patient had pCR or downstaging, an additional 10 patients were planned to be treated.

The primary analysis was of safety and included all patients who received at least one dose of the investigational regimen. The secondary endpoint of pCR or downstaging rate was estimated along with two-sided 95% confidence intervals (CIs) with the exact method of Clopper-Pearson intervals. The sample size of 20 in phase 2 part was determined by feasibility and relative precision of estimate.

**Supplementary Literature**

1. Rosen LS, Hurwitz HI, Wong MK, Goldman J, Mendelson DS, Figg WD, Spencer S, Adams BJ, Alvarez D, Seon BK, Theuer CP, Leigh BR, Gordon MS (2012) A phase I first-in-human study of TRC105 (Anti-Endoglin Antibody) in patients with advanced cancer. Clin Cancer Res 18:4820-4829. doi: 1078-0432.CCR-12-0098 [pii]

10.1158/1078-0432.CCR-12-0098

2. Shiozaki K, Harada N, Greco WR, Haba A, Uneda S, Tsai H, Seon BK (2006) Antiangiogenic chimeric anti-endoglin (CD105) antibody: pharmacokinetics and immunogenicity in nonhuman primates and effects of doxorubicin. Cancer Immunol Immunother 55:140-150. doi: 10.1007/s00262-005-0691-4

3. Gehan EA (1961) The determinatio of the number of patients required in a preliminary and a follow-up trial of a new chemotherapeutic agent. J Chronic Dis 13:346-353.
