# Supplementary material for: A phase I/II study of preoperative letrozole, everolimus, and carotuximab in stage 2 and 3 hormone receptor-positive and Her2-negative breast cancer
Source: Breast Cancer Res Treat. 2023 Feb 3;198(2):217–29. doi: 10.1007/s10549-023-06864-9 (PMC10020303; doi:10.1007/s10549-023-06864-9)
Supplement: Supplementary file 2 — Supplementary file2 (PPTX 102 kb) [file 10549_2023_6864_MOESM2_ESM.pptx]

## Slide 1
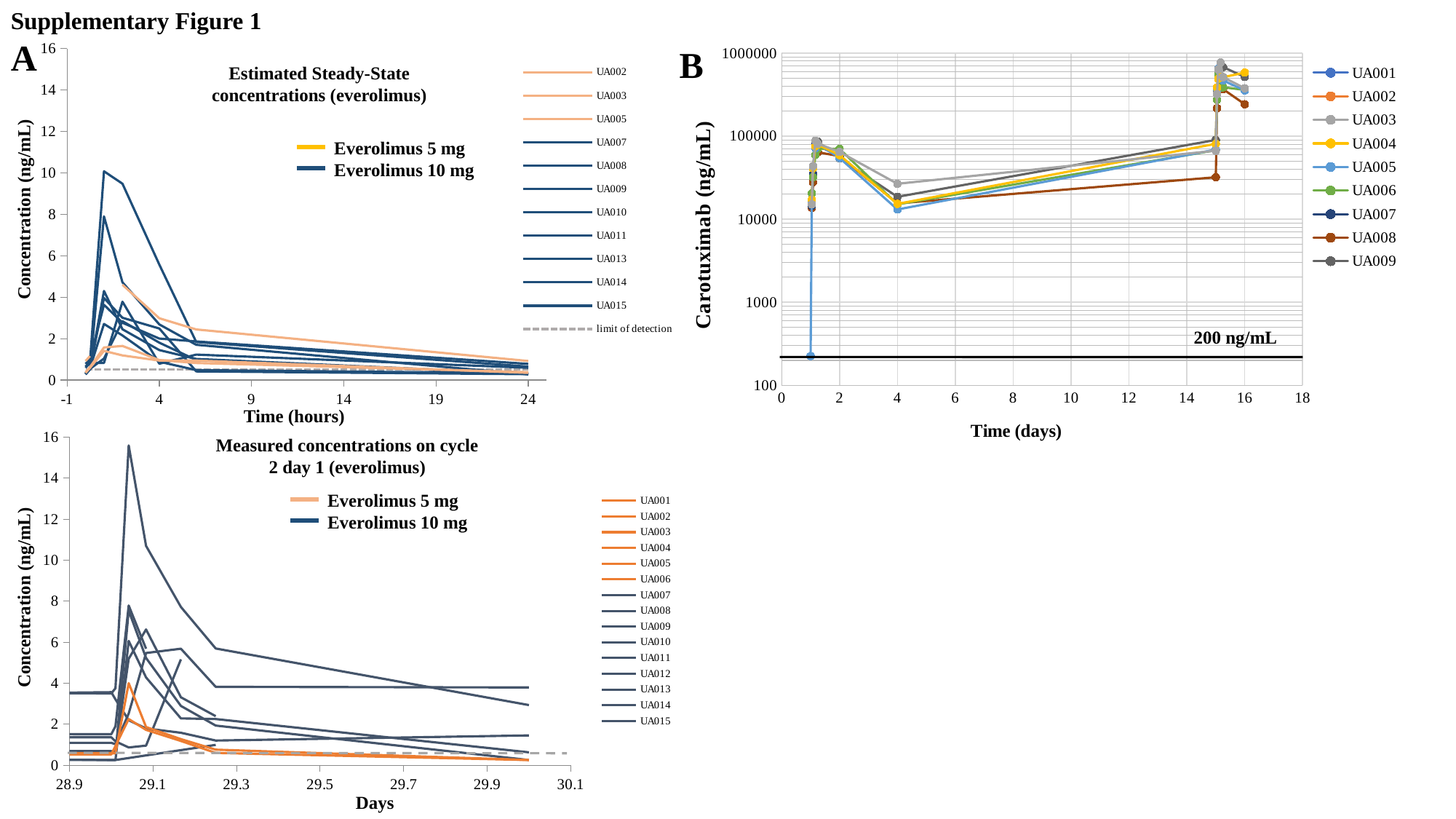

Supplementary Figure 1
A
### Chart
| Category | UA002 | UA003 | UA005 | UA007 | UA008 | UA009 | UA010 | UA011 | UA013 | UA014 | UA015 | limit of detection |
|---|---|---|---|---|---|---|---|---|---|---|---|---|B
### Chart
| Category | UA001 | UA002 | UA003 | UA004 | UA005 | UA006 | UA007 | UA008 | UA009 |
|---|---|---|---|---|---|---|---|---|---|Estimated Steady-State concentrations (everolimus)
Everolimus 5 mg
Everolimus 10 mg
Concentration (ng/mL)
200 ng/mL
Time (hours)
### Chart
| Category | time | UA001 | UA002 | UA003 | UA004 | UA005 | UA006 | UA007 | UA008 | UA009 | UA010 | UA011 | UA012 | UA013 | UA014 | UA015 |
|---|---|---|---|---|---|---|---|---|---|---|---|---|---|---|---|---|Measured concentrations on cycle 2 day 1 (everolimus)
Everolimus 5 mg
Everolimus 10 mg
Concentration (ng/mL)
Days
