# Supplementary material for: A phase I/II study of preoperative letrozole, everolimus, and carotuximab in stage 2 and 3 hormone receptor-positive and Her2-negative breast cancer
Source: Breast Cancer Res Treat. 2023 Feb 3;198(2):217–29. doi: 10.1007/s10549-023-06864-9 (PMC10020303; doi:10.1007/s10549-023-06864-9)
Supplement: Supplementary file 3 — Supplementary file3 (PPTX 38056 kb) [file 10549_2023_6864_MOESM3_ESM.pptx]

## Slide 1
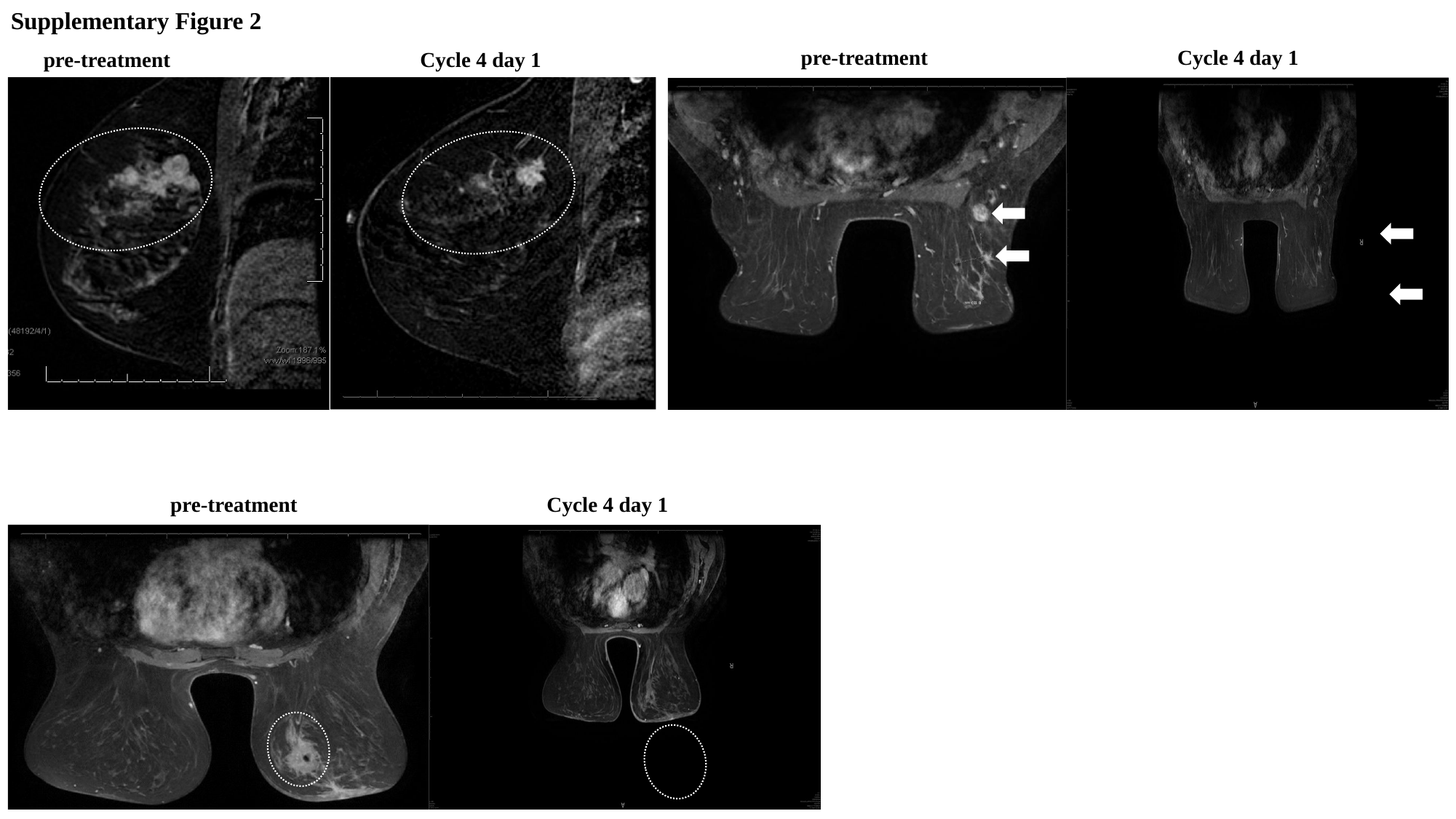

Supplementary Figure 2
pre-treatment
Cycle 4 day 1
pre-treatment
Cycle 4 day 1
pre-treatment
Cycle 4 day 1
