# Supplementary figures and images for: A phase I/II study of preoperative letrozole, everolimus, and carotuximab in stage 2 and 3 hormone receptor-positive and Her2-negative breast cancer
Source: Breast Cancer Res Treat. 2023 Feb 3;198(2):217–29. doi: 10.1007/s10549-023-06864-9 (PMC10020303; doi:10.1007/s10549-023-06864-9)

## Slide 1
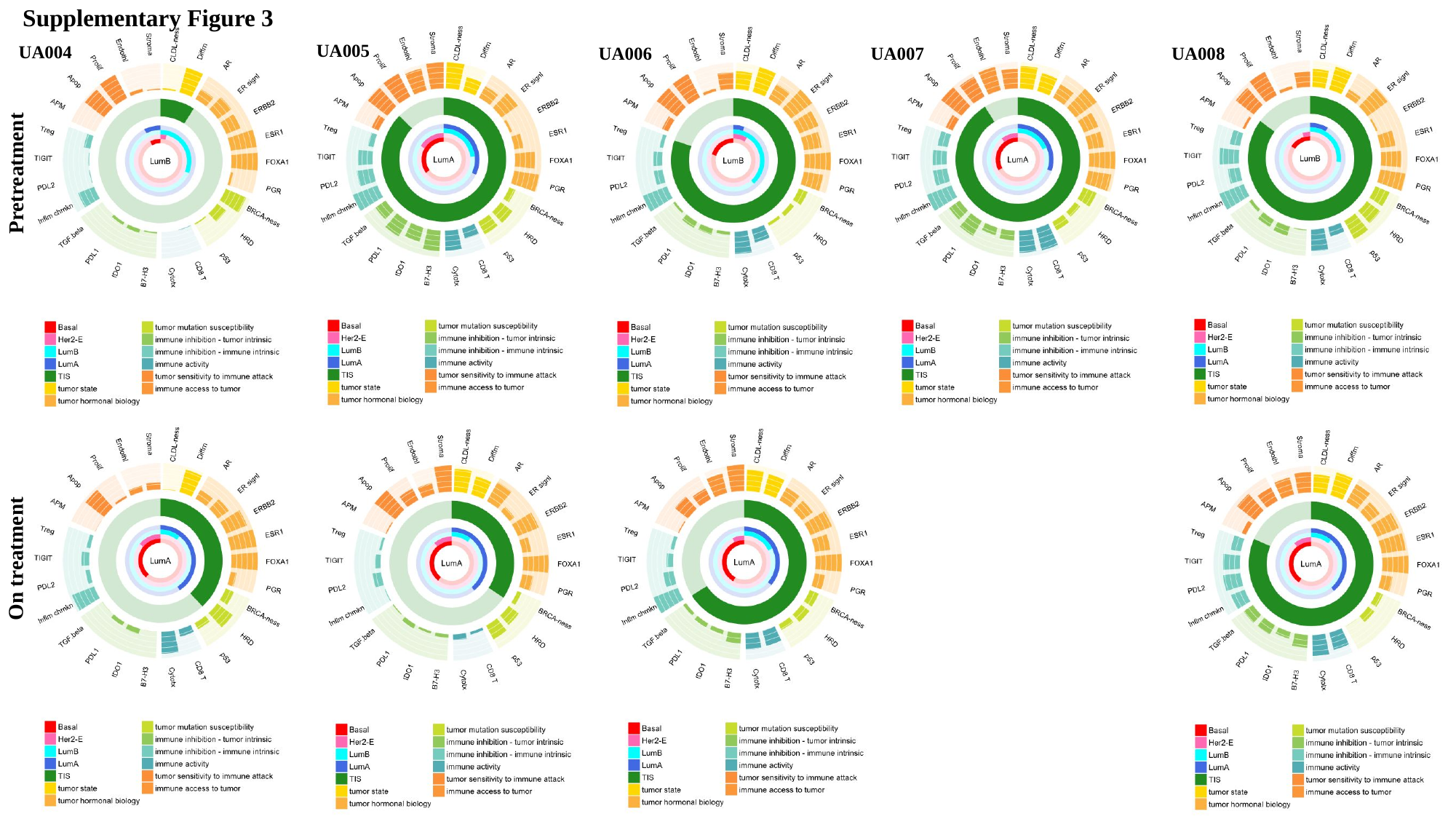

Supplementary Figure 3
UA005
UA004
UA006
UA008
UA007
Pretreatment
On treatment

Supplement: Supplementary file 4 — Supplementary file4 (PPTX 879 kb) [file 10549_2023_6864_MOESM4_ESM.pptx]

## Slide 1
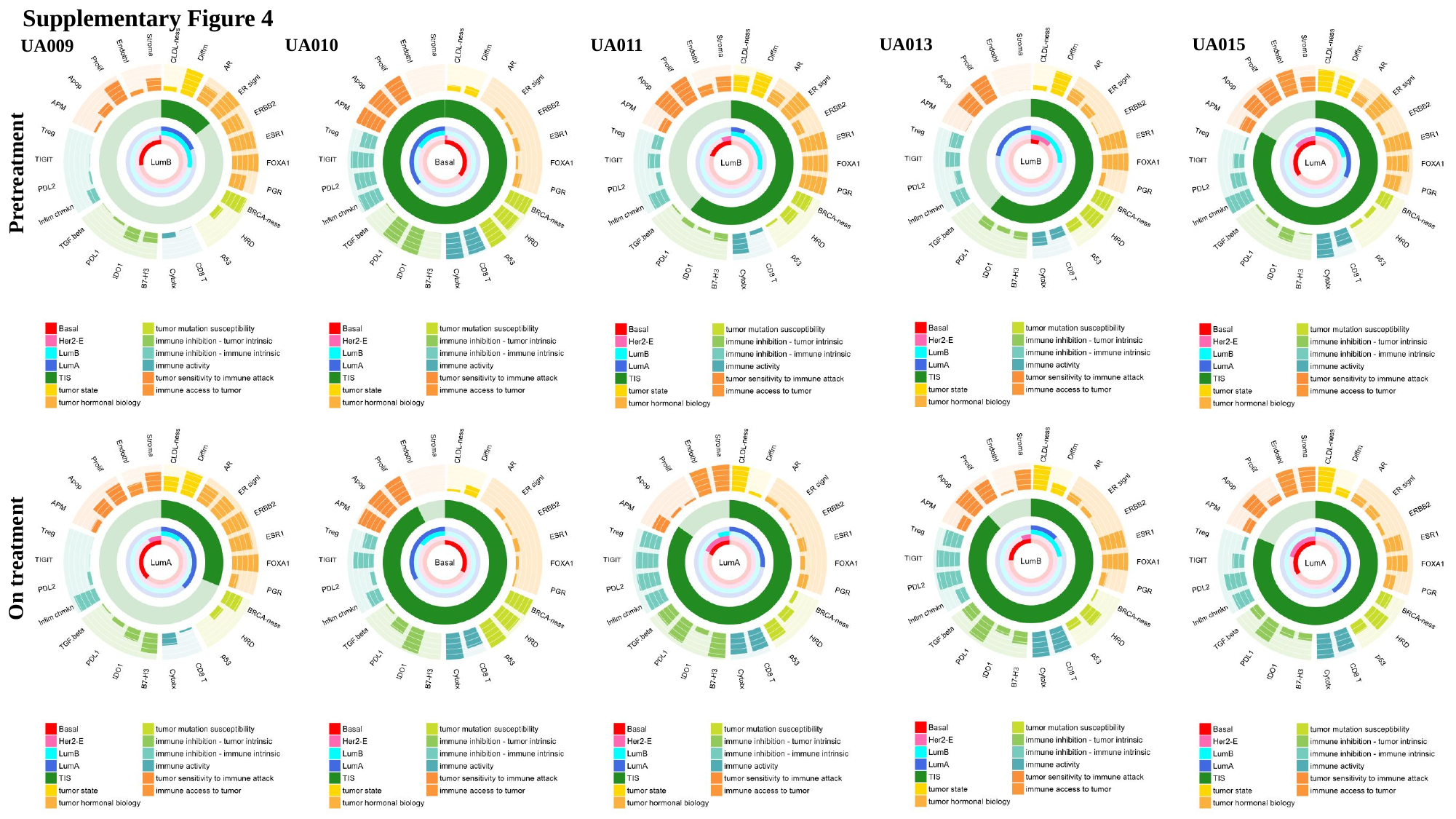

Supplementary Figure 4
UA013
UA015
UA010
UA011
UA009
Pretreatment
On treatment

Supplement: Supplementary file 5 — Supplementary file5 (PPTX 970 kb) [file 10549_2023_6864_MOESM5_ESM.pptx]

## Slide 1
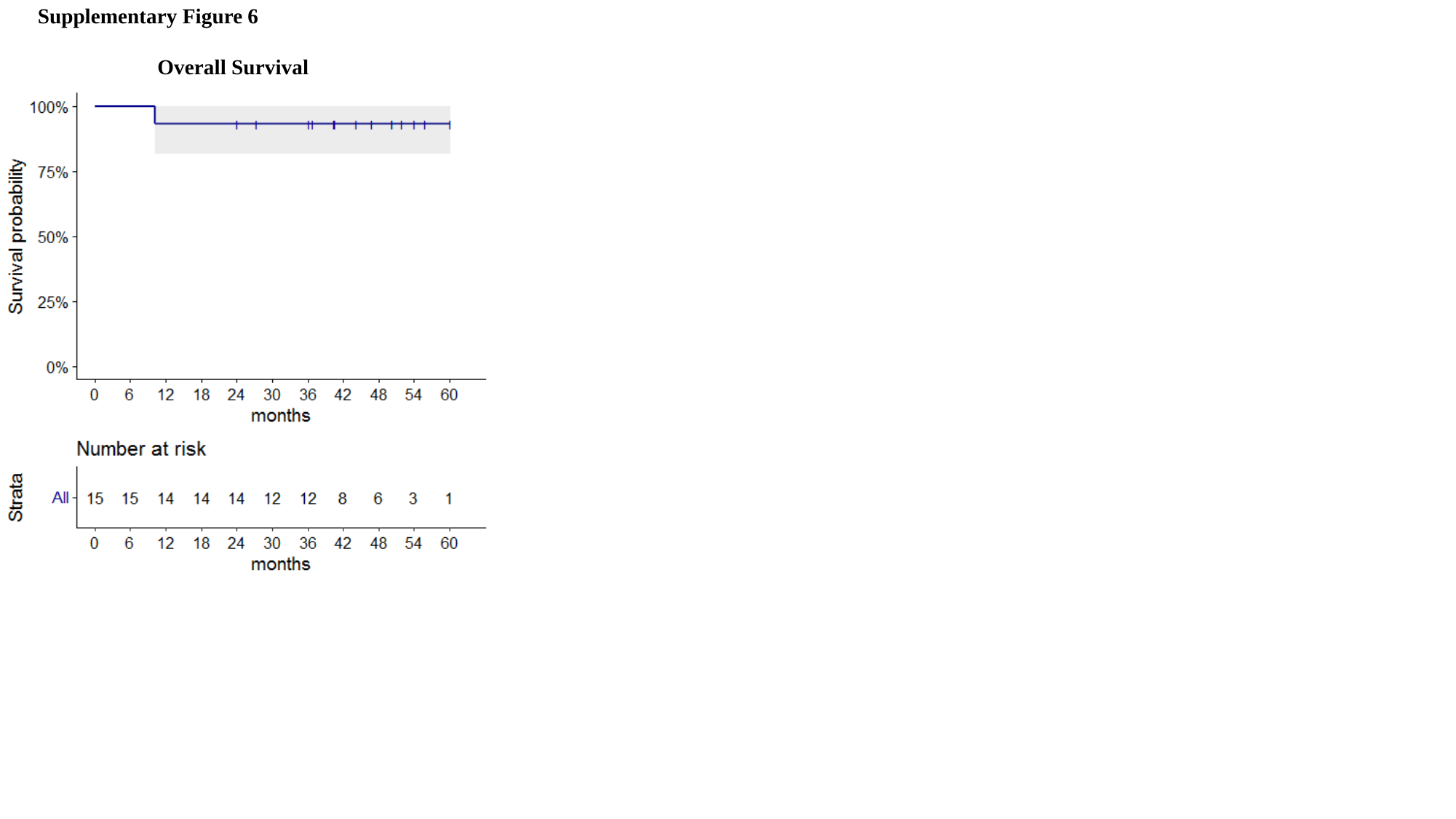

Supplementary Figure 6
Overall Survival

Supplement: Supplementary file 7 — Supplementary file7 (PPTX 52 kb) [file 10549_2023_6864_MOESM7_ESM.pptx]
