# Supplementary material for: A phase I/II study of preoperative letrozole, everolimus, and carotuximab in stage 2 and 3 hormone receptor-positive and Her2-negative breast cancer
Source: Breast Cancer Res Treat. 2023 Feb 3;198(2):217–29. doi: 10.1007/s10549-023-06864-9 (PMC10020303; doi:10.1007/s10549-023-06864-9)
Supplement: Supplementary file 6 — Supplementary file6 (PPTX 141 kb) [file 10549_2023_6864_MOESM6_ESM.pptx]

## Slide 1
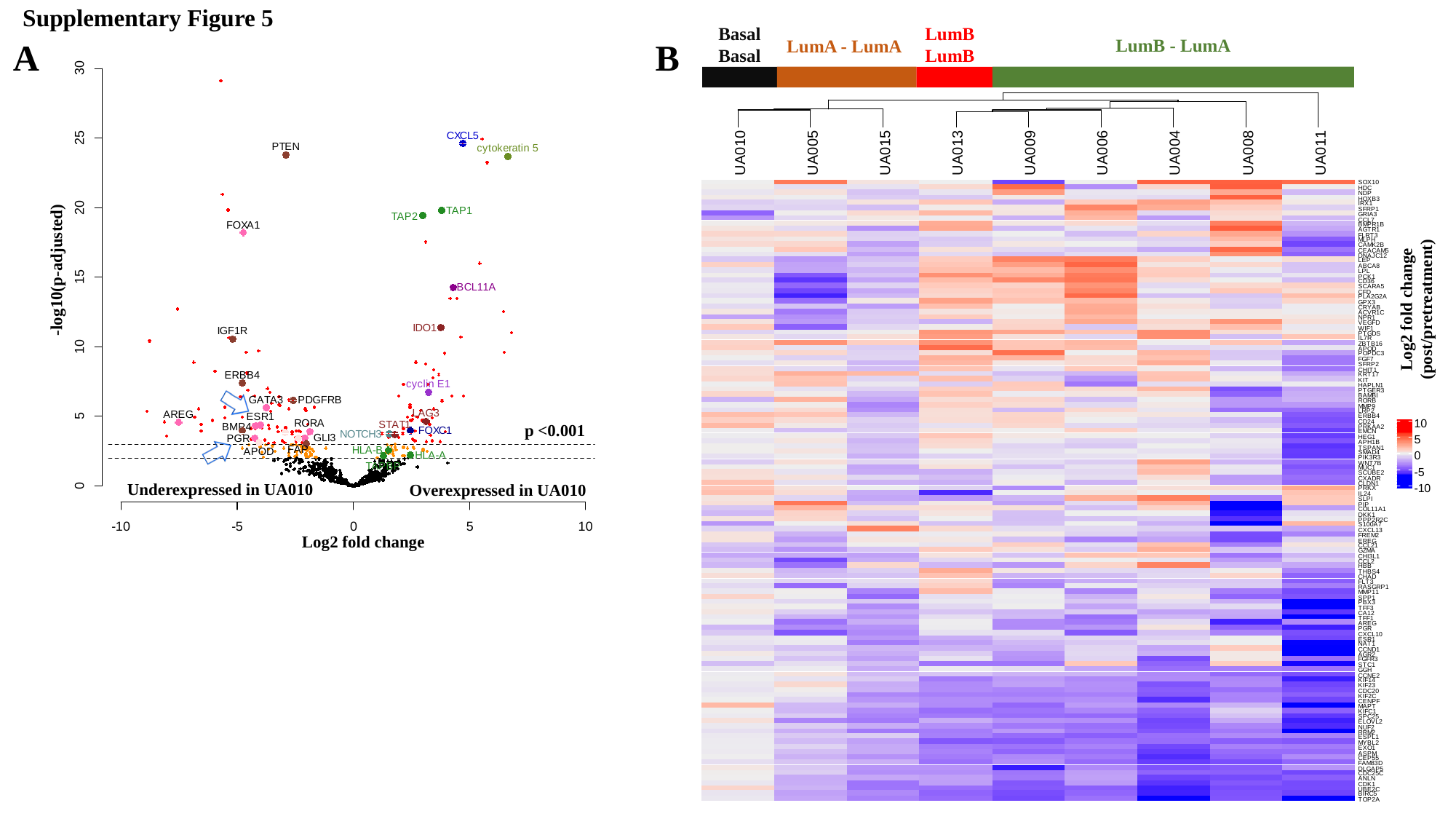

Supplementary Figure 5
Basal
Basal
LumB LumB
LumB - LumA
LumA - LumA
A
B
-log10(p-adjusted)
Log2 fold change (post/pretreatment)
p <0.001
Underexpressed in UA010
Overexpressed in UA010
Log2 fold change
