# Supplementary material for: A phase I/II study of preoperative letrozole, everolimus, and carotuximab in stage 2 and 3 hormone receptor-positive and Her2-negative breast cancer
Source: Breast Cancer Res Treat. 2023 Feb 3;198(2):217–29. doi: 10.1007/s10549-023-06864-9 (PMC10020303; doi:10.1007/s10549-023-06864-9)
Supplement: Supplementary file 8 — Supplementary file8 (DOCX 15 kb) [file 10549_2023_6864_MOESM8_ESM.docx]

**Supplementary table 1.** Pharmacokinetic analyses of everolimus and letrozole. Units of measurement in parentheses. For all parameters the geometric mean and geometric coefficient of variation (in parentheses) are provided except Tmax (median (range)) and half-life (arithmetic mean (standard deviation)).

|  | Everolimus (mg) | | Letrozole |
| --- | --- | --- | --- |
| Dose (n) | 5 (n=6) | 10 (n=9) | 2.5 (n=15) |
| AUC_0 to 24 hours_ (ng*h/mL) | 15.4 (41.9) | 20.9 (50.4) | 524 (33.6) |
| AUC_0 to infinity_ (observed) (ng*h/mL) | 23.7 (63) | 26 (45) | 1020 (25.0) |
| AUC_0 to infinity_ (predicted) (ng*h/mL) | 23.7 (63) | 25.8 (46) | 1020 (24.8) |
| AUC_672 to 696 hours_ (ng*h/mL) | 22.5 (39.3) | 32.1 (153) | 2400 (143) |
| Cmax_0 to 24 hours_ (ng/mL) | 1.56 (51.5) | 3.7 (55.5) | 39.9 (35.7) |
| Cmax_672 to 696 hours_ (ng/mL) | 2.63 (27.9) | 5.38 (86.7) | 217 (56.2) |
| Cmin_672 to 696 hours_ (ng/mL) | 0.448 (60.7) | 0.713 (109) | 92.5 (49.5) |
| Tmax (hours) | 2 (1-6) | 1 (1-4) | 2.00 (1-24) |
| C_trough_ (predose concentration, ng/mL) | 0.354 (58.3) | 0.323 (40.2) | 16.4 (54.3) |
| Non-compartmental half life (hours) | 13.4 (1.64) | 11.8 (7.86) | 24.7 (9.46) |
| Oral Clearance (observed, dose/AUC) (L/hour) | 211 (63000) | 385 (45000) | 2.44 (25000) |
| Oral Clearance (predicted, dose/AUC) (L/hour) | 211 (63000) | 388 (46000) | 2.44 (24800) |
| Clearance (L/day) | 4610 (CI 95%, 940) | 7974 (CI 95%, 706) | 44 (CI 95%, 14) |
| Mean Residence Time (observed)_0 to 24 hours_ (hours) | 19.1 (8.57) | 13.9 (51.2) | 33.9 (37.8) |
| Mean Residence Time (predicted)_0 to 24 hours_ (hours) | 19.1 (8.55) | 13.6 (53) | 33.9 (37.6) |
| Steady-state volume of distribution (observed) (L) | 4040 (71400) | 5360 (83200) | 82.9 (23300) |
| Steady-state volume of distribution (predicted) (L) | 4040 (71400) | 5290 (83400) | 82.9 (23300) |
| Volume of distribution (observed) (L) | 4060 (74400) | 5710 (85900) | 81.8 (24200) |
| Volume of distribution (predicted) (L) | 4060 (74300) | 5760 (85500) | 81.8 (24400) |
| Elimination rate constant (h^-1^) | 0.0520 (12.0) | 0.0673 (55.0) | 0.0299 (39.0) |

**Glossary: AUC,** area under the concentration-time curve; **Cmax,** maximum concentration; **Cmin,** minimum concentration; **C_trough_,** predose concentration.
