# Supplementary material for: A phase I/II study of preoperative letrozole, everolimus, and carotuximab in stage 2 and 3 hormone receptor-positive and Her2-negative breast cancer
Source: Breast Cancer Res Treat. 2023 Feb 3;198(2):217–29. doi: 10.1007/s10549-023-06864-9 (PMC10020303; doi:10.1007/s10549-023-06864-9)
Supplement: Supplementary file 9 — Supplementary file9 (DOCX 14 kb) [file 10549_2023_6864_MOESM9_ESM.docx]

**Supplementary table 2.** Pharmacokinetic analyses of carotuximab. Units of measurement in parentheses. Estimates and confidence intervals (calculated by 1000 bootstrap iterations).

|  | Carotuximab | |
| --- | --- | --- |
|  | 3 mg/kg (cycle 1 day 1) | 15 mg/kg (cycle 1 day 15) |
| Non-compartmental half-life (days) | 1.26 (95% CI, 1.1 – 1.55) | 12.33 (95% CI, 11.44 – 20.62) |
| AUC_0 to infinity_ (mg*day/mL) | 0.175 (95% CI, 0.162 – 0.194) | 1.949 (95% CI, 1.352 – 8.578) |
| AUMC_0 to infinity_ (mg*day/mL) | 0.319 (95% CI, 0.257 – 0.435) | 34.658 (95% CI, 23.026 – 207.242) |
| Mean residence time (days) | 1.82 (95% CI, 1.58 – 2.24) | 17.78 (95% CI, 16.51 – 29.75) |
| Clearance (dose/ AUC_0 to infinity_) (ml/kg/h) | 3.566 (95% CI, 3.225 - 3.869) | 0.321 (95% CI, 0.073 - 0.462) |
| Tmax (hours), median (range) | 4 (4 - 6) | 3 (0-24) |
| Estimated Volume of Distribution^a^ (mL/kg) | 155.8 | 137 |

AUMC, Area under the first moment curve.

^a^estimation made using the mean clearance and mean residence time
