# Supplementary material for: A phase I/II study of preoperative letrozole, everolimus, and carotuximab in stage 2 and 3 hormone receptor-positive and Her2-negative breast cancer
Source: Breast Cancer Res Treat. 2023 Feb 3;198(2):217–29. doi: 10.1007/s10549-023-06864-9 (PMC10020303; doi:10.1007/s10549-023-06864-9)
Supplement: Supplementary file 10 — Supplementary file9 (DOCX 28 kb) [file 10549_2023_6864_MOESM10_ESM.docx]

SUPPLEMENTARY FIGURE LEGENDS

**Supplementary Fig. 1**

**A** Estimated steady-state (upper panel, same as figure 1C) and measured on cycle 2 day 1 (lower panel) concentrations of everolimus. Estimated steady state and measured on cycle 2 day 1 concentrations of everolimus are concordant

**B** Carotuximab concentrations cycle 1 day 1 through cycle 1 day 16

**Supplementary Fig. 2**

Representative radiographic (MRI) responses. Note the decreased enhancement of the target lesion(s) indicative of decreased tumor vascularity

**Supplementary Fig. 3 and 4**

Intrinsic subtype classification and pathway analysis of the 10 patients with available paired samples. Note the classification of one immunophenotypically hormone receptor-positive and Her2 negative tumor as basal-like. The on-treatment sample for UA007 did not pass quality control

**Supplementary Fig. 5**

**A** Volcano plot of the differentially expressed genes between the basal-like/immunophenotypically HR+/Her2- (UA010) and luminal tumors. Functional annotation of the genes overexpressed in UA010 identified genes of the MHC class I antigen processing and presentation category (*TAP2*, *TAP1*, *HLA-A*, *HLA-B*, *TAPBP,* shown in green) overrepresented (p adjusted by the Bergamini method 0.006). The immune checkpoint components *LAG3*, *IDO1*, and the correlated *STAT1* (which have been associated with resistance to endocrine therapies in luminal B breast cancer[49]) were also differentially overexpressed in UA010. Other significantly differentially overexpressed genes of interest included *cytokeratin 5* (a defining immunohistochemical biomarker of the core basal phenotype[45, 46]), *BCL11A* and *FOXC1* (transcription factors typically overexpressed and driving tumor development, progression, invasion, and migration in triple-negative breast cancer[47, 48]), *cyclin E* (a key regulatory protein controlling the G_1_–S-phase transition whose overexpression of the low molecular weight isoform is associated with primary resistance to endocrine therapies[50]), *CXCL5* and *NOTCH3* (a chemokine that induces metastatic skeletal colonization[60] and a receptor that promotes metastatic seeding[61], respectively). On the other hand, the expression of genes involved in estrogen receptor signaling (*FOXA1, ESR1, PDGFRB, AREG, PTEN*, shown in pink) and the estrogen and progesterone receptor themselves (empty light blue arrow) were significantly downregulated in UA010.

**B** Unsupervised heatmap of gene expression changes with the investigational therapy. Tumor clustering by gene expression changes aligns with molecular subtype conversion or lack thereof (upper panel; Lum, luminal). Note the small magnitude of gene expression changes in the basal-like/immunophenotypically HR+/Her2- (UA010) tumor with the investigational therapy

**Supplementary Fig. 6**

Kaplan-Meier curve of overall survival. The patient with the basal-like/immunophenotypically HR+/Her2- breast cancer experienced an early relapse that was refractory to intensive chemotherapy and 4 months later passed away
